# Calculating and Comparing the Annualized Relapse Rate and Estimating the Confidence Interval in Relapsing Neurological Diseases

**DOI:** 10.3389/fneur.2022.875456

**Published:** 2022-06-10

**Authors:** Tetsuya Akaishi, Tadashi Ishii, Masashi Aoki, Ichiro Nakashima

**Affiliations:** ^1^Department of Education and Support for Regional Medicine, Tohoku University Hospital, Sendai, Japan; ^2^Division of General Medicine, Tohoku University Hospital, Sendai, Japan; ^3^Department of Neurology, Tohoku University Graduate School of Medicine, Sendai, Japan; ^4^Department of Neurology, Tohoku Medical and Pharmaceutical University, Sendai, Japan

**Keywords:** annualized relapse rate (ARR), confidence interval, F-distribution, Poisson distribution, generalized linear model (GLM), person-years method

## Abstract

Calculating the crude or adjusted annualized relapse rate (ARR) and its confidence interval (CI) is often required in clinical studies to evaluate chronic relapsing diseases, such as multiple sclerosis and neuromyelitis optica spectrum disorders. However, accurately calculating ARR and estimating the 95% CI requires careful application of statistical approaches and basic familiarity with the exponential family of distributions. When the relapse rate can be regarded as constant over time or by individuals, the crude ARR can be calculated using the person-years method, which divides the number of all observed relapses among all participants by the total follow-up period of the study cohort. If the number of relapses can be modeled by the Poisson distribution, the 95% CI of ARR can be obtained by finding the 2.5% upper and lower critical values of the parameter λ as the mean. Basic familiarity with F-statistics is also required when comparing the ARR between two disease groups. It is necessary to distinguish the observed relapse rate ratio (RR) between two sample groups (sample RR) from the unobserved RR between their originating populations (population RR). The ratio of population RR to sample RR roughly follows the F distribution, with degrees of freedom obtained by doubling the number of observed relapses in the two sample groups. Based on this, a 95% CI of the population RR can be estimated. When the count data of the response variable is overdispersed, the negative binomial distribution would be a better fit than the Poisson. Adjusted ARR and the 95% CI can be obtained by using the generalized linear regression models after selecting appropriate error structures (e.g., Poisson, negative binomial, zero-inflated Poisson, and zero-inflated negative binomial) according to the overdispersion and zero-inflation in the response variable.

## Introduction

The annualized relapse rate (ARR) is among the most reported indices in clinical studies of chronic relapsing diseases such as multiple sclerosis (MS) and neuromyelitis optica spectrum disorders (NMOSD) (1–4). In many recent clinical studies that enroll patients with demyelinating diseases in the central nervous system (CNS), crude or adjusted ARR is calculated and compared between disease groups and treatment groups to assess relapse activity by the respective diseases and treatments (3, 5–7). Although the calculation of ARR is relatively simple when the follow-up period is equal among all participants, careful consideration is required when calculating ARR in a cohort with different follow-up periods (5, 8). The calculation of ARR in a cohort with different follow-up periods is facilitated by the concept of the person-years method (9–11), which is one of the easiest and most common ways of calculating crude ARR. In addition to calculating ARR, the 95% confidence interval (CI) should be reported, but the correct ways of estimating the range differ by the distributional patterns of the relapse count data. In these processes, researchers need to be familiar with the general concept of the Poisson and negative binomial distributions, both of which are discrete distributions that express the probability of a specific number of events occurring under some specific situations. Generally, these distributions of the exponential family are used upon estimating the 95% CI, whereas the F-distribution is applied when comparing ARR between different treatment groups. Besides, the generalized linear model (GLM) will be applied to calculate the adjusted ARR and estimate its 95% CI, after selecting the appropriate error structure according to the distribution of the response variable. This report summarizes the basic concepts and simple methods for calculating crude and adjusted ARRs and estimating the 95% CIs, along with the prerequisites for fitting each distribution of the exponential family to the obtained data.

## Methods

### Prerequisites Before Calculating ARR and Estimating CI

When we calculate ARR from a patient group based on the total observed events and follow-up periods, the person-years method (or person-time method) is among the easiest and most common methods. However, this method requires the relapse rate not to significantly differ between participants and throughout the follow-up period. Consequently, if the cohort is comprised of patients with remarkably different relapse frequencies or apparent time-dependent changes in relapse activity, a simple application of the person-years method may be inappropriate. In such cases, a conceivable way to manage the individual-based or time-dependent differences in relapse activity may be to stratify the data according to the influential background (e.g., individuals, treatment, disease duration, relapse history, or disease stages). Another conceivable way would be utilizing GLM to calculate the adjusted ARR as described in the following sections.

### Concept of Poisson Distribution

The probability mass function of a Poisson distribution, with the discrete variable *k* denoting the number of observed occurrences (*k* = 0, 1, 2, …), is given as follows:


$$\begin{array}{l} f_{poisson}\left(k,\;\lambda \right)=\mathrm{Pr}\left(X=k\right)\;=\;\frac{\lambda^{k}e^{-\lambda }}{k!}, \end{array}$$

where the distribution parameter λ is equivalent to the expected number of events (X) in a fixed length of time, typically obtained from previously accumulated data. Because of the characteristics of the Poisson distribution, the parameter λ is also equal to the variance of the probability mass function of *f*(*X*, λ). Here, we consider the frequency of event occurrence following a Poisson distribution with parameter λ during a specific time period *t*. The expected number of events occurring during the time period 5*t* can be regarded as following the Poisson distribution with a parameter of 5λ. However, this is only the case if λ is known based on past accumulated data. In most clinical studies, λ is initially unknown, hence the need to estimate it together with the 95% CI from the sample data. When estimating the CI, Poisson distributions with different values of λ (mean and variance) are considered to determine the critical values of λ for a two-tailed test with α = 0.05. However, as a presumption of a Poisson distribution, the expectation [*E*(*X*)] and variance [*Var*(*X*)] are assumed to equal with λ, and Poisson distributions may underfit to overdispersed count data. Selecting inappropriate error structures for sample data may result in incorrect predictions of the 95% CI for ARR and linear predictor function upon using GLMs. Thus, when the sample variance of the number of event occurrences is found to be significantly larger than the sample mean (known as overdispersion), other distributions of the exponential family may be better fitting models. The test to determine the presence of overdispersion can be performed using R Statistical Software (R Foundation, Vienna, Austria) with the “dispersiontest” function of the AER package. The package can be installed by running each of the following R scripts:

>> install.packages(“AER”)
>> library(AER)
>> *GLM object name* < -glm(formula = ^*****^, data = *data frame name*, family = Poisson)
>> dispersiontest(*GLM object name*)

As the error structures, normal distribution is supposed in the classical linear regression models and binomial distribution is supposed in logistic regression models, but these distributions are usually not good fits for counting data like the numbers of relapses. Rather than these, the negative binomial distribution would be a better alternative fit as described in the next section. When there are too many data with the outcome event occurrence of zero (i.e., no relapse during the observation period), zero-inflated Poisson regression or zero-inflated negative binomial regression would better fit than normal Poisson or negative binomial regression. Zero-inflated models require installing the package “pscl” with “zeroinfl” function when using the R Statistics.

### Concept of Negative Binomial Distribution

**Figure 1:**
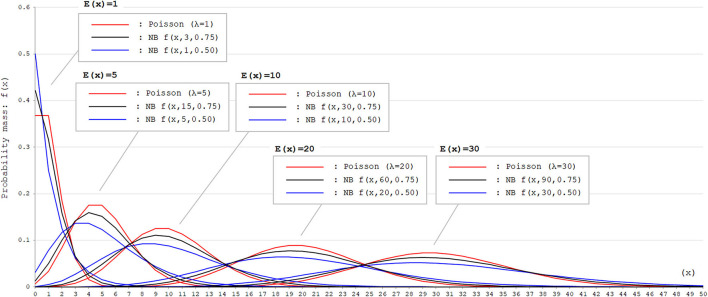
Poisson and negative binomial distributions with different parameters. Poisson and negative binomial (NB) distributions with different parameters that generate *E*(*x*) = 1, 5, 10, 20, *and* 30 are shown. The function for NB distributions [*f*(*x, k, p*)] denotes the probability mass of *x* failures by the time of *k* successes in trials with the successing probability of *p* in each attempt. This can be also expressed as *X* ~ *NB*(*k, p*). As can be seen, negative binomial distribution can produce distributions with larger variance (dispersion) than Poisson distribution with appropriate parameters. In Poisson distributions, the expected values [*E*(*x*)] correspond to the rate parameter λ. In NB distributions, *E*(*x*) corresponds to $\frac{k\left(1-p\right)}{p}$.

When the count data of relapses is found to be overdispersed, a conceivable good fit would be the negative binomial distribution. The general formula of the distribution is shown below, using the expectation μ (mean) and size parameter θ:


$$\begin{array}{l} \mathrm{Pr}\left(X=k\right)\;=\;\frac{\Gamma \left(\theta +k\right)}{k!\;\Gamma \left(\theta \right)}{\left(\frac{\theta }{\mu +\theta }\right)}^{\theta }{\left(\frac{\mu }{\mu +\theta }\right)}^{k}\\ \Gamma \left(\theta \right)\;=\;\int_{0}^{\infty }t^{\theta -1}e^{-t}dt \end{array}$$

where *X* is counting *k* failures that occur for a given number of θ successes. Then, the part of $\frac{\theta }{\left(\mu +\theta \right)}$ in the above formula corresponds to the probability of success (*p*) in each trial. With the negative binomial distribution, the skewness of the probability mass function can be modified by adjusting the size parameter θ. Thus, the negative binomial distribution is a good fit for dataset with event occurrence outcomes that appears right-skewed with high probability mass for event occurrence with 0. To visually confirm the difference in the distributions of Poisson and negative binomial distributions, these two distributions with different parameters are shown in Figure 1. As can be seen, when the expected values [*E*(*x*)] are equal, negative binomial distributions with appropriate parameters can express distributions with larger variance (dispersion) than the Poisson distribution.

### Comparing ARR Between Groups and Calculating Rate Ratio

When comparing the ARR between two groups with different diseases or treatments, a relapse rate ratio (RR) and its 95% CI can be obtained by using the F-distribution. Let the total follow-up period in each of the two groups (Group A and Group B) be denoted as {*T*_*A*_, *T*_*B*_}, and the observed total number of relapses in each group be denoted as {*N*_*A*_, *N*_*B*_}. Using these values, a sample RR can be defined as follows:


$$\begin{array}{l} \text{Sample RR}=\;\frac{Sample\;ARR\;in\;group\;A}{Sample\;ARR\;in\;group\;B}\;=\;\frac{\frac{N_{A}}{T_{A}}}{\frac{N_{B}}{T_{B}}} \end{array}$$

Next, we assume that the actual population ARR in each group, denoted as λ_*A*_ and λ_*B*_, are already known. The actual population ratio between the two groups can then be described as follows:


$$\begin{array}{l} \text{Population RR}=\;\frac{\lambda_{A}}{\lambda_{B}} \end{array}$$

If the number of observed events (i.e., *N*_*A*_ and *N*_*B*_) is sufficiently large, the ratio of $\left(\frac{Population\;RR}{Sample\;RR}\right)$ is known to approximately follow the F-distribution with 2*N*_*A*_ and 2*N*_*B*_ degrees of freedom (12, 13).


$$\begin{array}{l} \frac{Population\;RR}{Sample\;RR}\;\sim \;F\left(2N_{A},\;2N_{B}\right) \end{array}$$

For reference, the probability density function of the F-distribution with 2*N*_*A*_ and 2*N*_*B*_ degrees of freedom is expressed as follows:


$$\begin{array}{l} f\left(F\right)=\frac{{\left(\frac{N_{A}}{N_{B}}\right)}^{N_{A}}\cdot F^{\left(N_{A}-1\right)}}{B\left(N_{A},N_{B}\right)\;\cdot \;{\left(1+\frac{N_{A}}{N_{B}}F\right)}^{\left(N_{A}+N_{B}\right)}}\\ B\left(N_{A},N_{B}\right)=\int_{0}^{1}t^{\left(N_{A}-1\right)}\;\cdot \;{\left(1-t\right)}^{\left(N_{B}-1\right)}dt \end{array}$$

The peak, mean, and variance of an F-distribution can change according to the degrees of freedom; however, the formula below is always true and serves as a characteristic of F-distributions:


$$\begin{array}{l} {f\left(N_{A},\;N_{B}\right)}_{\alpha }\;=\;\frac{1}{{f\left(N_{B},\;N_{A}\right)}_{1-\alpha }}, \end{array}$$

where *f*(_*N*_*A*_, *N*_*B*_)α_ are random variables following *F*(*N*_*A*_, *N*_*B*_), for which the corresponding upper or lower cumulative probability (e.g., 0.025 or 0.05) is equivalent to α. Thus, the same results with the F-test upon comparing ARR between two groups can be obtained even after exchanging sample data between the two groups. These are the bases to obtain the RR between two groups and its 95% CI.

### GLM for Obtaining the Adjusted ARR

In clinical studies with relapsing diseases, it is important to adjust for baseline differences in critical covariates that are not dealt with by randomization (3, 5, 6). To counter this problem, it is important to obtain the adjusted ARR, in addition to the aforementioned crude ARR. Adjusted ARR is the ARR adjusted for several critical covariates, such as age, sex, disease duration, baseline disease severity, relapse activity in the last several years, activity on brain MRI, and the status of relapse preventions. When the dependent variable is measurement data like examination scores or body weight, a multiple regression analysis may be utilized. Or, when the dependent variable is binary data like survived/not survived, a logistic regression analysis may be useful. Meanwhile, upon calculating the adjusted ARR and estimating its 95% CI, the dependent variable is a count data of the number of relapses during a specific length of time. Generally, some distributions of the exponential family, such as Poisson distribution or negative binomial distribution, are known to be a good fit for counting data. GLM allows these exponential families to be used as the error structure. GLM is comprised of the following three important parts: (1) linear predictor (linear combination of response variable and independent variables), (2) link function, and (3) error structure (as probability distribution for the response variable). The general structure of GLM can be described as below with *Y*_*i*_ as the response variable and (*X*_1, *i*_, *X*_2, *i*_, …, *X*_*k, i*_) as the explanatory variables from an individual (*i*):


$$\begin{array}{l} g\left(\lambda_{i}\right)\;=\;b_{0}+\;b_{1}X_{1,\;i}+\;b_{2}X_{2,\;i}+\ldots +\;b_{k}X_{k,\;i}\\ Y_{i}\;\sim \;Error\;structure\;with\;parameter\;\lambda_{i}\;\left(e.g.,\;Poission\left(\lambda_{i}\right)\right) \end{array}$$

where *g*^−1^ is the link function (i.e., logarithmic function in Poisson and negative binomial). For example, the Poisson regression model can be expressed as below:


$$\begin{array}{l} \lambda_{i}\;=\exp\left(b_{0}+\;b_{1}X_{1,\;i}+\;b_{2}X_{2,\;i}+\ldots +\;b_{k}X_{k,\;i}\right)\\ Y_{i}\;\sim \;\frac{{\lambda_{i}}^{Y_{i}}e^{-\lambda_{i}}}{Y_{i}!} \end{array}$$

By searching for a coefficient (slope) of each explanatory variable that maximizes the following log-likelihood L, the parameters (*b*_0_, *b*_1_, *b*_2_, …, *b*_*k*_) can be determined:


$$\begin{array}{l} L\left(b\right)\;=\\ \overset{n}{\underset{i=1}{\sum }}\log\left(p\left(Y_{i}|g\left(b_{0}+\;b_{1}X_{1,\;i}+\;b_{2}X_{2,\;i}+\ldots +\;b_{k}X_{k,\;i}\right)\right)\right) \end{array}$$

Then, the adjusted ARR, based on GLM like Poisson regression or negative binomial regression models, can be obtained, together with the 95% CI.

## Results

### ARR Based on Person-Years Method

**Figure 2:**
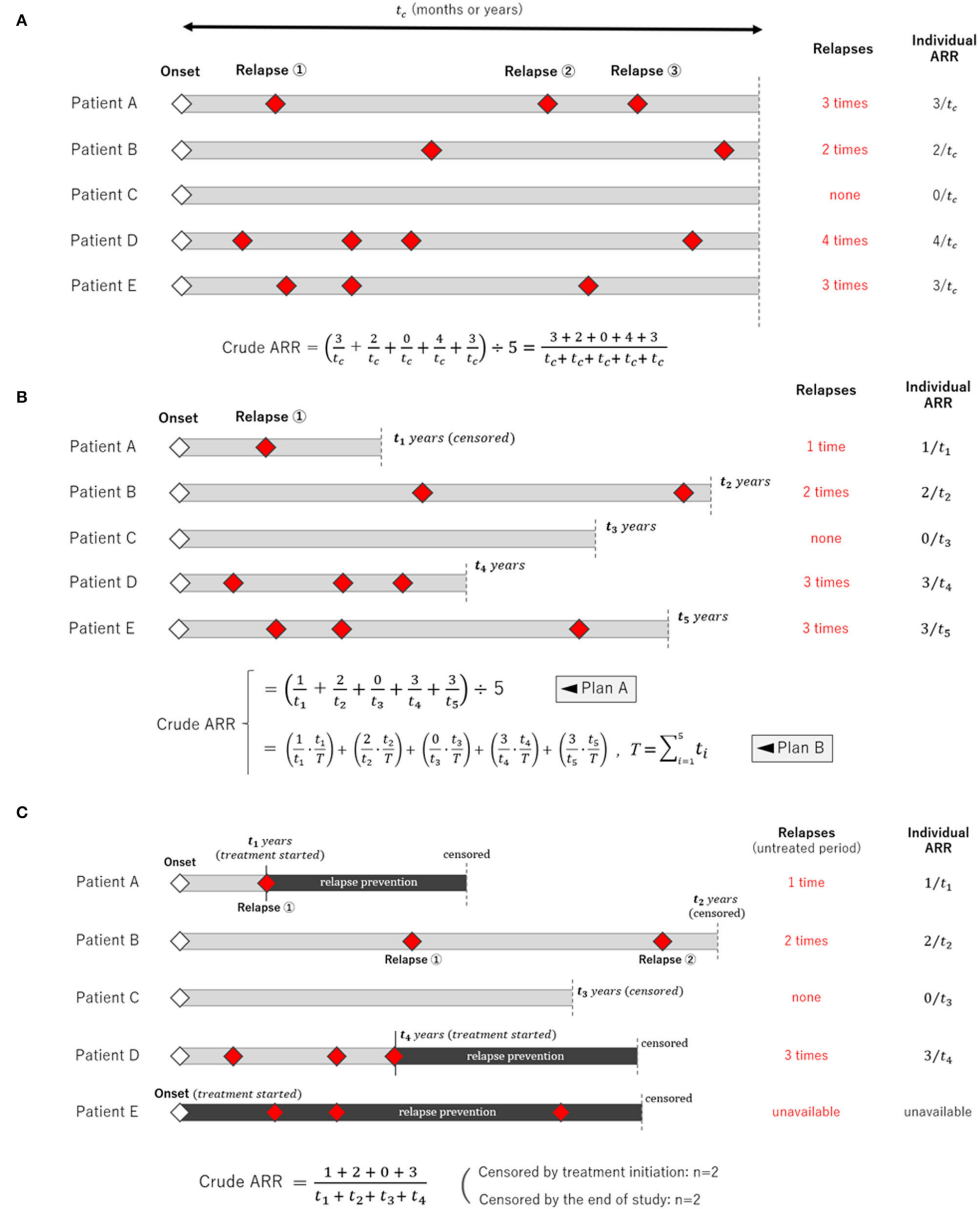
Schemes of clinical studies with different types of the follow-up period. Three different scenarios of clinical observational studies with different follow-up periods for each participant are shown. **(A)** The follow-up period was fixed and equal among the study participants. **(B)** The follow-up period varied among the participants, in which case averaging only the individual relapse rate (Plan A) is insufficient, and applying the person-years method (Plan B) is more appropriate. This is because the person-years method can weigh different follow-up periods in each participant. **(C)** The follow-up period in many participants is censored by the occurrence of an event, and each participant's follow-up period could be influenced by the occurrence of an event.

Calculating the crude (unadjusted) ARR is simple when the follow-up period is equal among study participants, as shown in Figure 2A. Most clinical trials that use ARR as a primary endpoint, such as those enrolling patients with relapsing demyelinating diseases in the CNS, correspond to this case as far as the number of dropout cases is negligibly small. In this scenario, averaging the individual-based ARR yields the same result as the person-years calculation; thus, both are appropriate. However, the situation differs when the follow-up periods differ between the study participants, as shown in Figure 2B. In this case, two methods for calculating the ARR can be conceived: averaging the individual-based ARR (Plan A) or calculating using the person-years method (Plan B) (12). In Plan A, the individual weighting to account for the different follow-up periods was totally ignored. In contrast, Plan B applies the procedure of weighted summing adjusted for each participant's follow-up period (i.e., $\frac{t_{i}}{T}$). The general formula for Plan B is expressed as follows:


$$\begin{array}{l} \text{ARR}=\;\underset{i}{\sum }\left(\frac{r_{i}}{t_{i}}\;\times \;\frac{t_{i}}{T}\right)\;=\;\frac{\sum_{i}r_{i}}{\sum_{i}t_{i}}. \end{array}$$

In the above formula, the subscript *i* represents each individual, *r*_*i*_ is the number of relapses during follow-up in each individual, *t*_*i*_ is the length of follow-up in each individual, and *T* is the total sum of follow-up periods within the cohort. This formula illustrates the general concept of the person-years method for calculating ARR. To ensure accurate results, we recommend checking in advance before using the person-years method whether the prerequisites listed in the Methods section (i.e., relapse frequency does not differ between participants, by time, or by preceding relapse status) can be regarded to be fulfilled.

### Crude ARR Before Starting Relapse Prevention

Upon calculating AAR, additional careful consideration is required when the observation follow-up periods are censored at the time of event occurrence, as shown in Figure 2C. For example, this occurs when a clinical study evaluates ARR during the untreated period before initiating long-term relapse prevention treatments, which typically begin immediately following a relapse (e.g., relapse prevention in neuromyelitis optica spectrum disorder, anticonvulsant in epilepsy, and antiplatelet in stroke). A conceivable approximation can be obtained by simply applying the aforementioned person-years method for the time period before starting treatments. However, strictly speaking, the obtained ARR will be somewhat higher than the actual rate unless the observed number of events in each participant is sufficiently large enough for the truncated fractions of the follow-up period to be negligible. Therefore, it is preferable for each report to record the number of cases that were censored by the end of the study, and specify those who were censored by the initiation of relapse prevention immediately after relapses. With this information, the reported data will be interpreted more correctly.

### Estimating 95% CI of ARR

Here, a disease is considered in which the number of event occurrences (*X*) during the follow-up period of *T* person-years follows the Poisson distribution of the unknown mean λ. If a clinical study observed *k* event occurrences during *T* person-years, the 95% CI for λ will be expressed as follows using the probability mass function of the Poisson distribution (*f*_*poisson*_(*X*, λ)):


$$\begin{array}{l} \lambda_{-\;\left[95\%\;CI\right]}\;\leq \;\lambda \;\leq \;\lambda_{+\;\left[95\%\;CI\right]}\\ \overset{\infty }{\underset{X=k}{\sum }}f_{poisson}\left(X,\;\lambda_{-\;\left[95\%\;CI\right]}\right)\;=0.025\\ \overset{k}{\underset{X=0}{\sum }}f_{poisson}\left(X,\;\lambda_{+\;\left[95\%\;CI\right]}\right)\;=0.025 \end{array}$$

where

λ_−[95*% CI*]_: 2.5% lower critical value for the observation of *k* events
λ_+[95*% CI*]_: 2.5% upper critical value for the observation of *k* events

To estimate the α% confidence interval with a Poisson distribution, the above formulae can be generalized as follows:


$$\begin{array}{l} \lambda_{-\;\left[\alpha \%\;CI\right]}\;\leq \;\lambda \;\leq \;\lambda_{+\;\left[\alpha \%\;CI\right]}\\ \overset{\infty }{\underset{X=k}{\sum }}f_{poisson}\left(X,\;\lambda_{-\;\left[\alpha \%\;CI\right]}\right)\;=\;\left(\frac{100-\alpha }{100}\right)\times \frac{1}{2}\\ \overset{k}{\underset{X=0}{\sum }}f_{poisson}\left(X,\;\lambda_{+\;\left[\alpha \%\;CI\right]}\right)\;=\;\left(\frac{100-\alpha }{100}\right)\times \frac{1}{2} \end{array}$$

This range can be easily determined using the R Statistical Software with the following script, where *X* denotes the number of observed event occurrences:

>> Poisson.test(*X*)

With *X* = *k* and the average number of event occurrences λ being the number during the follow-up period of *T* person-years, the 95% CI of the ARR ($=\frac{\lambda }{T}$) can be approximated as follows:


$$\begin{array}{l} \frac{\lambda_{-\left[95\%\;CI\right]}}{T}\;\leq \;\frac{\lambda }{T}\;\leq \;\frac{\lambda_{+\left[95\%\;CI\right]}}{T} \end{array}$$

**Figure 3:**
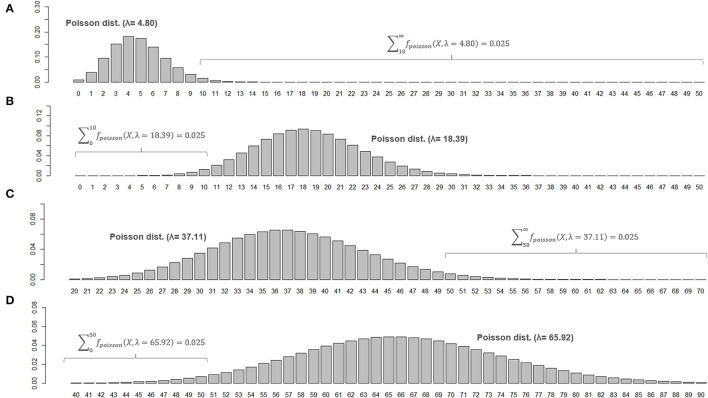
Poisson distributions of different means and 95% CIs for the parameter λ. Poisson distributions of different means that realize an upper or lower cumulative probability of 0.025, with the number of observed events (*X*) at 10 **(A,B)** or 50 **(C,D)**, are shown. To be noted, the ranges for horizontal axis are different between **(C,D)**.

**Figure 4:**
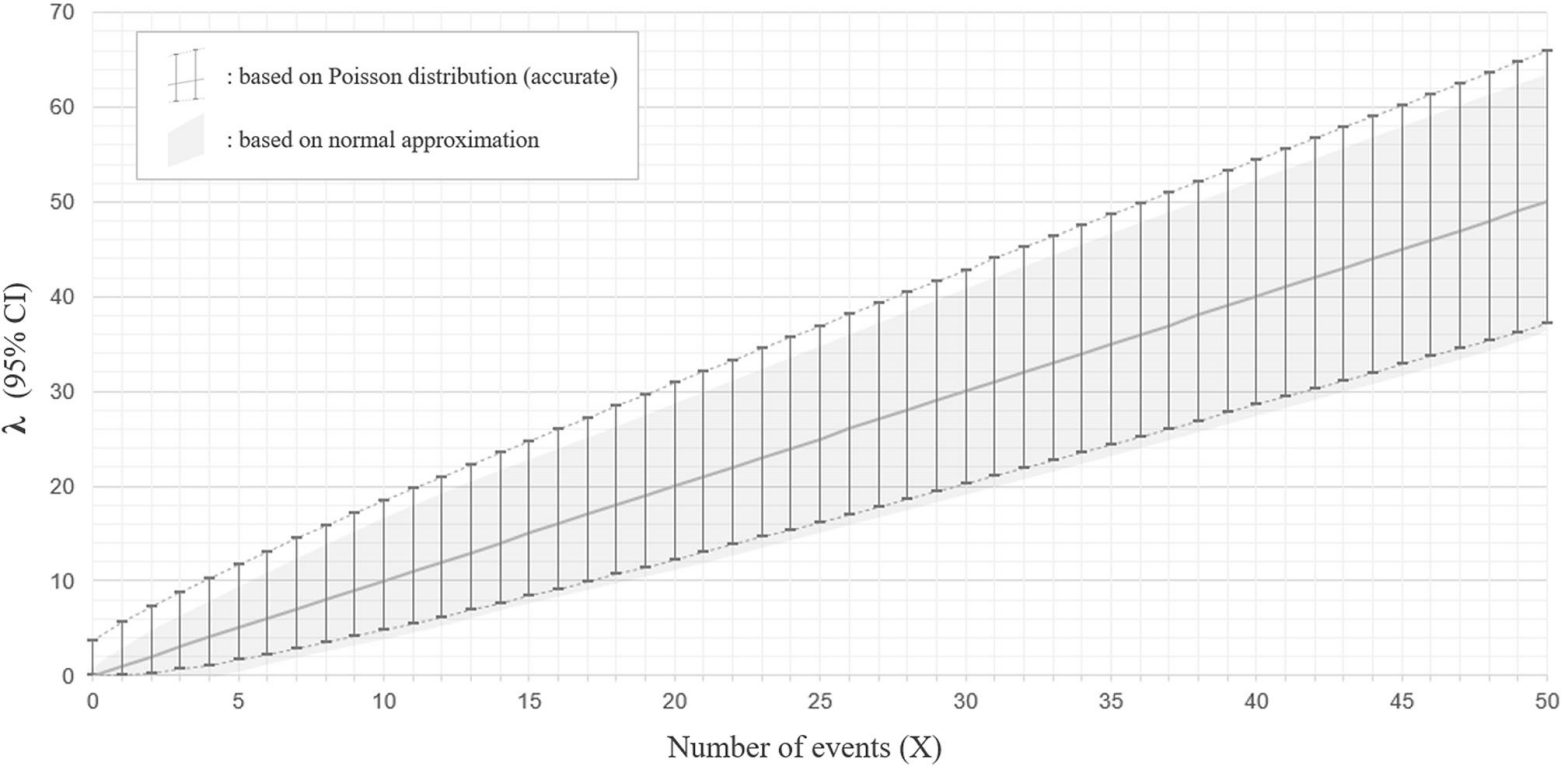
Ninety five percentage confidence interval for the parameter λ with Poisson distribution and with normal approximation. The estimated 95% confidence intervals (CIs) for the mean λ with a different number of observed events (*X*) between 0 and 50, based on the Poisson distribution (accurate version; error bars with broken lines) or normal approximation (approximate version; gray color filled area), are shown. The estimated CIs for mean λ are not bilaterally symmetrical from *X* with the accurate version, whereas they are bilaterally symmetrical from *X* with normal approximation.

When two studies yield the same crude ARR but involve different total follow-up periods, the estimated 95% CI will be different between them. Consider Case 1, where a total of 10 relapses (*X* = 10) were observed during 20 person-years of follow-up, and Case 2, where a total of 50 relapses (*X* = 50) were observed during 100 person-years of follow-up. In both cases, the crude ARR is equal to 0.50. For Case 1, the Poisson distributions with mean values 4.80 and 18.39 yield the upper and lower cumulative probability of 0.025, respectively (Figures 3A,B). As the total follow-up period was 20 person-years, the 95% CI approximation will be provisionally acquired as 0.24–0.92. In Case 2, the Poisson distributions with mean values 37.11 and 65.92 yield upper and lower cumulative probabilities of 0.025 (Figures 3C,D). As the follow-up period was 100 person-years, the 95% CI approximation is 0.37–0.66. Poisson distribution is not bilaterally symmetrical unless *X* is extremely large (i.e., normal approximation to Poisson distribution), and the estimated 95% CI of λ will also be bilaterally asymmetrical from *X* (Figure 4, error bars with broken lines).

With a sufficiently large number of observed relapses, a normal approximation of the Poisson distribution is possible (14). Then, the 95% CI of the average number of event occurrences (λ) can be approximated as follows:


$$\begin{array}{l} k-1.96\times \sqrt{k}\;\leq \;\lambda \;\leq k+1.96\times \sqrt{k}, \end{array}$$

where *k* is the number of events that occurred during the entire follow-up period of *T* for all participants. As a simplified estimate of the 95% CI of the ARR ($=\frac{\lambda }{T}$), the following inequality expression is also permissible:


$$\begin{array}{l} \frac{k-1.96\times \sqrt{k}}{T}\;\leq \;\frac{\lambda }{T}\;\leq \;\frac{k+1.96\times \sqrt{k}}{T}. \end{array}$$

As this method approximates the Poisson distribution to a normal distribution, it is not strictly accurate. The 95% CI ranges obtained by the Poisson distribution do not exactly overlap with those approximated by the normal distribution, especially for the upper critical values (Figure 4).

### Relapse Rate Ratio (RR) Between Two Disease Groups

The significance of the difference in RR between two groups can be evaluated by assuming the population RR to be exactly 1.0 (null hypothesis: *H*_0_). Then, the inverse number of the sample RR ($\frac{1}{Sample\;RR}$) can be regarded as roughly following *F*(2*N*_*A*_, 2*N*_*B*_). By fitting the calculated F-statistics ($\frac{1}{Sample\;RR}$) to the F-distribution with (2*N*_*A*_, 2*N*_*B*_) degrees of freedom, a one-tailed *p*-value is obtained as the upper or lower cumulative probability. Since statistical comparisons in most clinical studies are performed with a two-sided test, the *p*-value for rejecting the null hypothesis can be obtained by doubling the one-tailed *p*-value. For example, if the observed sample RR was 1.380 between two groups with 50 and 80 observed events, the calculated F-statistics based on ($\frac{1}{Sample\;RR}$) would be approximately 0.7246, which roughly follows *F*(100, 160). The lower cumulative probability at the cut-off level of *F* = 0.7246 is ~0.0406. Thus, the two-tailed *p*-value typically sought in clinical studies will be ~0.0812. Consequently, the null hypothesis (*H*_0_) that “the population RR is 1.0” cannot be rejected with an alpha level of 0.05. The same results were obtained even when groups A and B were exchanged, in which case the F-statistic based on ($\frac{1}{Sample\;RR}$) was 1.38, which roughly follows *F*(160, 100). The upper cumulative probability at the cutoff level of *F* = 1.380 was ~0.0406, which yields the same *p*-value of 0.0812.

The approximated 95% CI of the relapse rate ratio (RR) between two groups can be estimated using F-distribution with (2*N*_*A*_, 2*N*_*B*_) degrees of freedom, where {*N*_*A*_, *N*_*B*_} are the total number of relapses observed in each group. When {*N*_*A*_, *N*_*B*_} are sufficiently large (e.g., >10 events for each), the approximate (100−2α)% CI of the actual population RR can be estimated using the following expression from the observed sample RR:


$$\begin{array}{l} Sample\;RR\;\times \;F_{-}\left(2N_{A},\;2N_{B}\right)\;\leq \;Population\;RR\;\leq \;Sample\;RR\\ \times \;F_{+}\left(2N_{A},\;2N_{B}\right), \end{array}$$

where

*F*_−_(2*N*_*A*_, 2*N*_*B*_) : lower α% point of F with (2*N*_*A*_, 2*N*_*B*_) degrees of freedom
*F*_+_(2*N*_*A*_, 2*N*_*B*_) : upper α% point of F with (2*N*_*A*_, 2*N*_*B*_) degrees of freedom

For example, when determining the 95% CI of the RR, α = 2.5 will be applied to the above expression. In the example with a sample RR of 1.380 and sample sizes of 50 and 80, the upper and lower critical levels of 95% CI (i.e., the 2.5 and 97.5 percentiles) for the F distribution with 100 and 160 degrees of freedom are 0.696 and 1.416, respectively. Thus, the estimated 95% CI for the population RR is 0.96–1.95. As estimated from a *p*-value above 0.05 (i.e., 0.0812), the estimated 95% CI for the population RR includes 1.0.

### Different 95% CIs for ARR With Poisson vs. Negative Binomial

**Figure 5:**
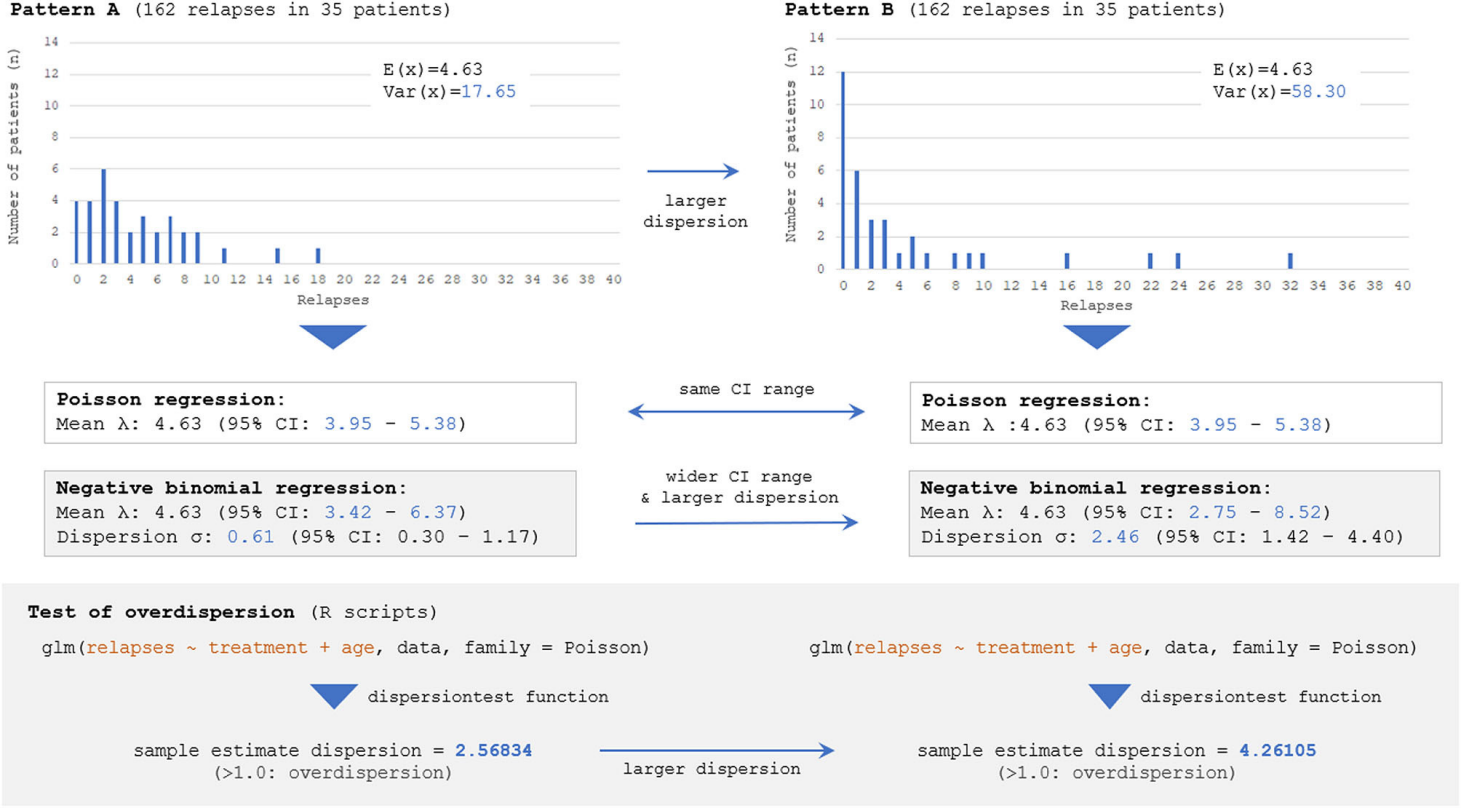
Estimated 95% CI with different dispersions of dataset by Poisson and negative binomial models. Two different distributions with the same numbers of overall events (162 total relapses as outcome measure) and participants (*n* = 35), but with different distributions of event frequency showing distinct dispersion levels [*V*(*X*)=17.65 for **Pattern A** vs. 58.30 for **Pattern B**], are considered. The follow-up period is regarded to be the same (e.g., 1 or 2 years) among 35 patients. The Poisson model can yield only a single 95% CI for both distributions because of the characteristics of the Poisson distribution, and the information regarding the skewness could not be accounted for. Meanwhile, negative binomial distributions can yield two distinct 95% CI for the two distributions, reflecting the different skewness. The overdispersion test by R Statistics revealed that the estimated dispersion levels from these two distributions were both larger than 1.0, suggesting that negative binomial models would be a better fit than Poisson for them.

Next, the influence of selecting incorrect error structures (Poisson vs. negative binomial) for an overdispersed count data on the estimated 95% CI range for ARR will be described. Here, two different datasets presenting different distributions of event frequency as the outcome measure, with exactly the same numbers of overall relapse observations (162 relapses) and enrolled patients (*n* = 35), are considered. The two distributions are shown in Figure 5; the left distribution is less dispersed with a smaller variance [*Var*(*x*)] than the right one, but both distributions have the same numbers of overall relapses and enrolled patients. As can be seen, fitting with Poisson distributions yielded the same 95% CI of the expectation [*E*(*x*)] with the two different distributions, whereas fittings with negative binomial distributions successfully yielded two distinct 95% CI ranges for the two distributions. This finding can be understood from the aforementioned background of Poisson distributions that the parameter λ (expectation or mean) is equal to the variance of the probability mass function. As far as the overall relapse time is constant, Poisson distributions can only produce a single 95% CI range even with different patterns of distribution. This fact emphasizes the importance of selecting a correct error structure with GLM according to the type and distribution of the response variable.

### Adjusted ARR

**Figure 6:**
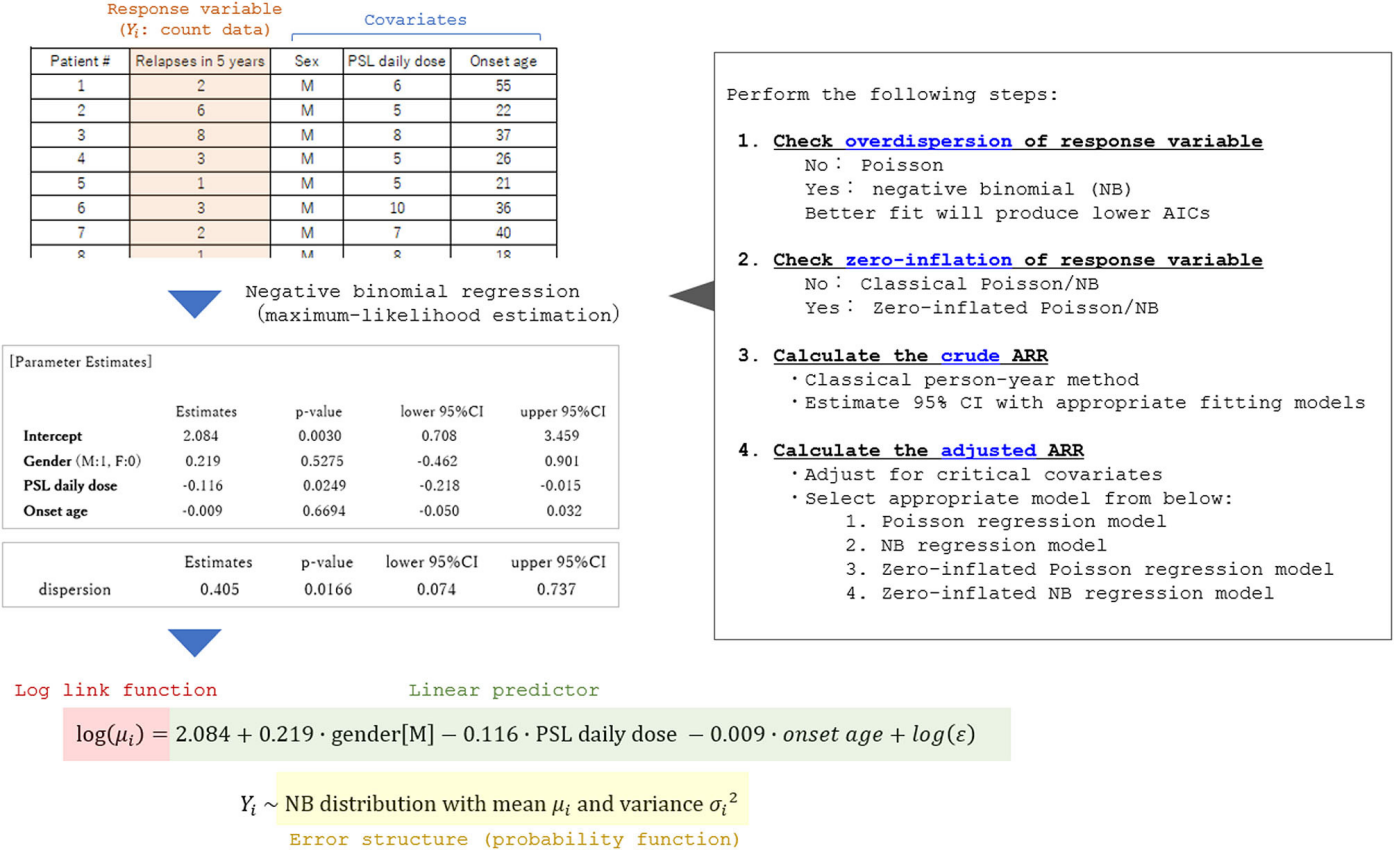
Process of applying the generalized linear model to calculate adjusted ARR. A fictitious dataset from 35 patients with a relapsing disease is used in the analysis. To select an appropriate general linear regression model, overdispersion and zero-inflation of the response variable (relapse count in the dataset) should be checked in advance. By performing the generalized linear regression analyses with appropriate error structures, the coefficient for each critical covariate in the linear predictor function can be obtained together with the 95% CI. By utilizing the obtained prediction expression, the expectation of the response variable adjusted for the covariates (typically set as the means of the whole population) can be estimated.

Currently, there is no established international guideline in the way of calculating the adjusted ARR. Most of the previous studies reporting adjusted ARR and the 95% CI utilized the negative binomial regression model (3, 5–7). Certainly, the Poisson regression model or negative binomial regression model is a promising way of obtaining the adjusted ARR adjusted for some critical covariates. An example of the process of obtaining adjusted ARR and the 95% CI is shown in Figure 6. The exponential of the shown estimate for intercept does not equal the so-called “adjusted ARR.” Rather, the value being derived from the intercept has the interpretation of the relapse rate of a fictitious individual with all covariates equal to zero both for the continuous and dummy variables. However, in actual cases, supposing a fictitious case with an onset age of 0 years is unrealistic. A simple conceivable solution would be substituting the means of the whole population for the explanatory variables in the obtained generalized linear regression model.

For example, a clinical study by Buron et al. reported the adjusted ARR and the 95% CI in patients with relapsing-remitting MS stratified by the type of relapse prevention (teriflunomide vs. dimethyl fumarate) is reviewed here (5). When discussing “adjusted” ARR, researchers often have a particular exposure of interest in their minds. The interest was the type of relapse prevention (teriflunomide vs. dimethyl fumarate) in the previous study. In their attempt to examine the predicted mean ARR for each treatment subgroup (*n* = 1,469 with teriflunomide and *n* = 767 with dimethyl fumarate), adjusted for the potential confounding variables, Buron et al. set the confounding variables as the mean values of the whole population (*n* = 2,236). By doing this, covariates between exposure groups can be balanced, and the adjusted ARR in each exposure group can be obtained and safely compared. SAS software can be used to obtain the 95% CI for the mean ARR upon substituting the mean values for the confounding variables, using SAS PROC GENMOD with the LSMEANS statement. In summary, when obtaining adjusted ARR and the 95% CI, the first step is to perform a Poisson or negative binomial regression model. The second step will be to obtain the mean ARR and 95% CI for each exposure of interest by setting the rest of the confounding variables as the mean values of the whole population.

### Comparing the Estimated ARR Between Different Approaches

Lastly, by using actual data from a previously published clinical study that enrolled patients with anti-aquaporin-4 antibody-positive NMOSD (8), the obtained ARR and the 95% CI are compared between the aforementioned different statistical approaches. The used cohort was comprised of 31 patients with NMOSD, who were not treated by relapse preventions during the observation period of 2 years from the onset. A total of 16 relapses were observed during the overall follow-up period of 62 person-years. This cohort had no particular exposure of interest, and the ARRs were obtained for the whole population. As potential covariates to calculate the adjusted ARR, data regarding the onset of age and administration of acute treatment with high-dose intravenous corticosteroid therapy were further collected. The average onset age was 39.9 years and the frequency of acute treatment was 0.42 in the whole population, and these values were substituted for the confounding variables to obtain the adjusted ARR. The obtained ARR (95% CI) for each of the introduced statistical approaches is listed in Table 1. As can be seen, the estimated 95% CI for ARR differed between the applied statistical approaches. Moreover, the expected means of ARR differed between unadjusted and adjusted ARR, according to the list of selected covariates.

**Table 1:** ARR and 95% CI with different statistical approaches.

	**ARR (95% CI)**	**Notes**
**Crude ARR** (person-time method; 95% CI by fitting Poisson distributions)	0.258 (0.148–0.419)	A total of 16 relapses during an overall follow-up of 62 person-years: crude ARR = 16/62 = 0.258. The 95% CI is simply estimated from the two values of “16” (relapses) and “62” (person-years).
**Crude ARR** (person-time method; 95% CI based on a normal approximation to the Poisson)	0.258 (0.132–0.385)	The 95% CI was approximated by using: $\frac{k-1.96\times \sqrt{k}}{T}\leq \frac{\lambda }{T}\leq \frac{k+1.96\times \sqrt{k}}{T}$, where *k* is the observed number of relapses, λ is the Poisson parameter, and *T* is the overall follow-up period [person-years]. Here, *k* is 16 (relapses) and *T* is 62 (person-years).
**Unadjusted ARR** (Poisson regression; not adjusted for covariates)	0.258 (0.151–0.406)	Poisson regression model is applied to the data of the number of relapses (*n =* 31). This method is applicable when the distribution is (1) not overdispersed and (2) without an excess of zero counts.
**Unadjusted ARR** (NB regression; not adjusted for covariates)	0.258 (0.143–0.445)	NB regression model is applied to the data of the number of relapses (*n =* 31). This method is applicable when the distribution is (1) overdispersed and (2) without an excess of zero counts.
**Unadjusted ARR** (zero-inflated Poisson regression; not adjusted for covariates)	0.303 (0.097–0.669)	Zero-inflated Poisson regression model is applied to the data of the number of relapses (*n =* 31). This method is applicable when the distribution is (1) not overdispersed and (2) with an excess of zero counts.
**Unadjusted ARR** (zero-inflated NB regression; not adjusted for covariates)	0.258 (0.000–0.636)	Zero-inflated NB regression model is applied to the data of the number of relapses (*n =* 31). This method is applicable when the distribution is (1) overdispersed and (2) with an excess of zero counts.
**Adjusted ARR** (Poisson regression; adjusted for age and acute treatment)	0.247 (0.145–0.421)^*^	Poisson regression model is applied to the data of the number of relapses (*n =* 31), adjusted for the onset age and administration of acute treatments after the onset.
**Adjusted ARR** (NB regression; adjusted for age and acute treatment)	0.246 (0.129–0.472)^*^	NB regression model is applied to the data of the number of relapses (*n =* 31), adjusted for the onset age and administration of acute treatments after the onset.
Incorrect application of Wald method for “proportion”	0.258 (0.166–0.379)	This is an incorrect approach with a misunderstanding that “16 successes out of 62 attempts,” not “16 relapses during 62 person-year follow-up.”

*The listed ARR and the 95% CI are obtained from data of 31 patients with anti-aquaporin-4 antibody-positive neuromyelitis optica spectrum disorders, who were not treated by relapse preventions during the observation period of 2 years after the onset. A total of 16 relapses were observed during the overall follow-up period of 62 person-years. Crude ARR and 95% CI can be obtained from the two values of “16” and “62.” Meanwhile, unadjusted/adjusted ARR and 95% CI, obtained by applying the generalized linear regression models, require raw data (n = 31) of relapse numbers from each patient*.

* *For calculating the adjusted ARR and 95% CI, the explanatory variables were set as the mean of the population (i.e., 39.9 years were substituted for the onset age and 0.42 were substituted for the administration of acute treatments)*.

*ARR, annualized relapse rate; CI, confidence interval; NB, negative binomial*.

## Discussion

In this report, the simple methods for calculating the crude ARR in a study group and approximating its 95% CI, as well as that of the relapse rate ratio between two study groups and the adjusted ARR, were outlined with specific examples. With the understanding of the general concepts and prerequisites for these statistical methods, the subsequent procedures can be performed using suitable calculation software, such as R Statistical Software. All of the statistical procedures described in this study can also be performed by using other statistical software such as SAS, SPSS, JMP, and Stata. As for the R Statistics, it should be noted that additional R packages such as AER, stats, MASS, and pscl are required to be installed in advance to perform overdispersion test, zero-inflation test, and multivariate GLMs. When the obtained count data are not overdispersed, the classical or zero-inflated Poisson model can be applied. When the obtained dispersion level by dispersion test is large enough to suggest an overdispersion, the classical or zero-inflated negative binomial distribution would be a better fit. Although the negative binomial distribution is an extended version of the Poisson distribution, it is not a generalized version of the Poisson. For count data without overdispersion, Poisson is a better fit of GLM than negative binomial in many cases. This can be known by checking the level of the obtained Akaike's Information Criterion to be lower with the Poisson regression model than that with the negative binomial regression model with such datasets.

When estimating the 95% CI of the ARR based on count data of relapse, careful attention is needed not to mistakenly estimate the CI for the population “proportion” based on the central limit theorem with binomial distribution. Consider a situation in which 50 relapses were observed during 100 person-years. As described in the Results section, the correct way to estimate the 95% CI for ARR involves applying a Poisson distribution to produce a bilaterally asymmetric CI with 0.371–0.659. In contrast, if the range is mistakenly estimated by applying the “Wald method” as described below to estimate the “proportion” (i.e., 50 successes in 100 attempts), the obtained 95% CI for the “proportion” will be 0.402–0.598:


$$\begin{array}{l} \hat{p}-z\times \sqrt{\frac{p\left(1-p\right)}{n}}\;\leq p\;\leq \;\hat{p}+z\times \sqrt{\frac{p\left(1-p\right)}{n}}, \end{array}$$

where *z* is the $\frac{\left(100-\alpha \right)}{2}$ % point of the standard normal distribution (15, 16). Rate and proportion are different mathematical concepts. When considering two phenomena A and B, rate measures the change in A (e.g., measurements or counts) against the change in B (e.g., time). Since ARR is a rate, it can theoretically take any positive integers or zero (i.e., 0 ≤ λ < ∞), when the criterion to define relapses regarding the minimum relapse interval is ignored. For example, if 200 relapses were observed during a 50-person-year follow-up period, the point estimation for $\hat{\lambda }$ is 4.0. Meanwhile, the proportion between the two phenomena is the ratio of B against A, where B is incorporated into A. When *k* patients out of a total of *n* (0 ≤ *k* ≤ *n*) have exhibited relapses during the follow-up period, the point estimation for the proportion of patients with relapses ($\hat{p}$) will be $\frac{k}{n}$, which must be always between 0 and 1 ($0\leq \hat{p}\leq \;1$).

Finally, to understand the general concept of statistical methods for estimating the 95% CI with focused parameters such as mean, rate, and ratio, it would be helpful to clearly distinguish whether the expressed parameters in the calculation process are for the observed samples (sample parameters; usually expressed with Latin alphabets) or for the unobserved populations (population parameters; usually expressed with Greek alphabets). In most clinical studies, sample parameters are available from the collected data. The main purposes of the statistical analyses in these studies include the estimation of population parameters, such as whole patient populations, ethnic groups, or the entire country, from the sample datasets. For example, when applying the Poisson distribution to estimate the 95% CI of ARR, the number of observed events (*X*) is a sample parameter and the estimated mean (λ) is the unknown population parameter, which the estimation aims to reach. In the case of 50 relapses observed during 100-person-years, the obtained crude ARR of 0.50 is a sample parameter and does not guarantee that the average population ARR is also 0.50.

A potential limitation of this study is the fact that ARR may not be the ideal primary endpoint in future clinical trials of MS, as interest has gradually shifted from suppressing the relapse rate to suppressing the chronic progression of neurological deterioration and brain atrophy (17). Moreover, some recent clinical studies and computer-based simulations that focus on the relapse frequency in MS utilized the time to first relapse, rather than ARR, as the primary endpoint (18–20). Nonetheless, ARR can be a promising primary endpoint for chronic relapsing diseases without progressive clinical deterioration free of relapses, such as NMOSD (8, 21, 22).

## Conclusion

Regardless of whether the follow-up period varies between study participants, the crude ARR can be obtained using the person-years method so long as the relapse rate does not remarkably vary between participants, the rate is not influenced by the time lapse from enrollment, and each participant's follow-up period is not influenced by the occurrence of an event. A 95% CI for ARR in each data series can be estimated by applying appropriate error structures (e.g., Poisson, negative binomial, zero-inflated Poisson, and zero-inflated negative binomial) according to the distributions of the count data for relapses, whereas a 95% CI for the ARR ratio between two data series can be estimated using an F-distribution with (2*N*_*A*_, 2*N*_*B*_) degrees of freedom. Adjusted ARR and the 95% CI can be obtained by using GLMs by selecting appropriate error structures and relevant sets of covariates to be adjusted for demographics, treatments, and baseline relapse activities.

## Data Availability Statement

The original contributions presented in the study are included in the article/supplementary material, further inquiries can be directed to the corresponding author/s.

## Author Contributions

TA analyzed the data and drafted the manuscript. TI, MA, and IN supervised the process of the study and critically revised the manuscript. All authors contributed to the article and approved the final version.

## Conflict of Interest

The authors declare that the research was conducted in the absence of any commercial or financial relationships that could be construed as a potential conflict of interest.

## Publisher's Note

All claims expressed in this article are solely those of the authors and do not necessarily represent those of their affiliated organizations, or those of the publisher, the editors and the reviewers. Any product that may be evaluated in this article, or claim that may be made by its manufacturer, is not guaranteed or endorsed by the publisher.
